# Force Control Deficits in Individuals with Parkinson’s Disease, Multiple Systems Atrophy, and Progressive Supranuclear Palsy

**DOI:** 10.1371/journal.pone.0058403

**Published:** 2013-03-11

**Authors:** Kristina A. Neely, Peggy J. Planetta, Janey Prodoehl, Daniel M. Corcos, Cynthia L. Comella, Christopher G. Goetz, Kathleen L. Shannon, David E. Vaillancourt

**Affiliations:** 1 Department of Applied Physiology and Kinesiology, University of Florida, Gainesville, Florida, United States of America; 2 Physical Therapy Program, Midwestern University, Downers Grove, Illinois, United States of America; 3 Department of Kinesiology and Nutrition, University of Illinois at Chicago, Chicago, Illinois, United States of America; 4 Department of Bioengineering, University of Illinois at Chicago, Chicago, Illinois, United States of America; 5 Department of Physical Therapy, University of Illinois at Chicago, Chicago, Illinois, United States of America; 6 Department of Neurological Sciences, University of Illinois at Chicago, Chicago, Illinois, United States of America; 7 Department of Neurological Sciences, Rush University Medical Center, Chicago, Illinois, United States of America; 8 Department of Neurology, University of Florida, Gainesville, Florida, United States of America; 9 Department of Biomedical Engineering, University of Florida, Gainesville, Florida, United States of America; Karolinska Institute, Sweden

## Abstract

**Objective:**

This study examined grip force and cognition in Parkinson’s disease (PD), Parkinsonian variant of multiple system atrophy (MSAp), progressive supranuclear palsy (PSP), and healthy controls. PD is characterized by a slower rate of force increase and decrease and the production of abnormally large grip forces. Early-stage PD has difficulty with the rapid contraction and relaxation of hand muscles required for precision gripping. The first goal was to determine which features of grip force are abnormal in MSAp and PSP. The second goal was to determine whether a single variable or a combination of motor and cognitive measures would distinguish patient groups. Since PSP is more cognitively impaired relative to PD and MSAp, we expected that combining motor and cognitive measures would further distinguish PSP from PD and MSAp.

**Methods:**

We studied 44 participants: 12 PD, 12 MSAp, 8 PSP, and 12 controls. Patients were diagnosed by a movement disorders neurologist and were tested off anti-Parkinsonian medication. Participants completed a visually guided grip force task wherein force pulses were produced for 2 s, followed by 1 s of rest. We also conducted four cognitive tests.

**Results:**

PD, MSAp, and PSP were slower at contracting and relaxing force and produced longer pulse durations compared to controls. PSP produced additional force pulses during the task and were more cognitively impaired relative to other groups. A receiver operator characteristic analysis revealed that the combination of number of pulses and Brief Test of Attention (BTA) discriminated PSP from PD, MSAp, and controls with a high degree of sensitivity and specificity.

**Conclusions:**

Slowness in contracting and relaxing force represent general features of PD, MSAp, and PSP, whereas producing additional force pulses was specific to PSP. Combining motor and cognitive measures provides a robust method for characterizing behavioral features of PSP compared to MSAp and PD.

## Introduction

Parkinson’s disease (PD) is characterized by bradykinesia, rigidity, tremor, and postural instability. Although these signs are routinely identified in a clinical exam, detecting additional features that differentiate PD from other forms of Parkinsonism can be more difficult. Atypical Parkinsonian disorders, such as the Parkinsonian variant of multiple system atrophy (MSAp) and progressive supranuclear palsy (PSP), can mimic signs of PD and the correct diagnosis may only become clear as the disease progresses [1], [2]. Clinical drug trials treating PD have mistakenly included patients with atypical Parkinsonism [1]. Further, correct diagnosis is important for the patient and caregiver to make decisions regarding treatment and long-term planning, and to establish coping and support mechanisms. It is therefore important to explore behavioral tests that may provide distinguishing characteristics [3].

Force control in healthy individuals has been well-characterized by the motor control literature. In healthy adults, the rate of force increase is dependent on the target force amplitude, such that the duration from force onset to peak force is relatively constant [4]. This characteristic is described by the pulse-height policy, wherein different force amplitudes are achieved by proportional scaling of the rate of force increase [5]. This is an important feature of force control because PD is characterized by a slow rate of force development [6], [7], [8], [9], [10] and force relaxation [8], [11], [12]. Levodopa increases the rate of force development and relaxation in patients with PD [11]. Less is known about the production of grip force in patients with atypical Parkinsonian disorders such as MSAp and PSP. Patients with MSAp have difficulty sequencing a grasp-to-lift task and exert greater forces following lift-off relative to healthy individuals [13], consistent with most studies of PD [7]. To the best of our knowledge, Muratori and colleagues’ work (10) is the only examination of grip force production in MSAp and no previous investigations have characterized grip force control in PSP.

The present study examined grip force control in healthy individuals and patients with PD, MSAp, and PSP. The first goal of the study was to determine which features of grip force are abnormal in MSAp and PSP. We hypothesized that similar to PD, patients with MSAp and PSP would be characterized by a slow rate of force development and force relaxation. Further, since bedside clinical tests have shown that PSP patients are unable to abruptly stop a repetitive movement [14], we hypothesized that PSP patients would have difficulty inhibiting the production of grip force pulses. In addition to examining force control, we examined several cognitive measures. Cognitive measures may provide an adjunct for distinguishing between movement disorders, especially since patients with PSP typically undergo faster decline in cognitive domains relative to patients with PD and MSAp [15]. The second goal of the study was to determine whether a single variable or a combination of motor and cognitive measures would distinguish atypical Parkinsonian patients from typical PD. Since PSP patients experience greater cognitive decline relative to patients with PD and MSAp, we hypothesized that a combination of motor and cognitive measures would distinguish patients with PSP from those with PD and MSAp.

## Methods

### Ethics Statement

All procedures were approved by the Institutional Review Board at the University of Illinois at Chicago and consistent with the Declaration of Helsinki. All procedures were carried out with the adequate understanding and written consent of the participants involved in the research.

### Participants

This study included 44 participants: 12 PD, 12 MSAp, 8 PSP, and 12 healthy controls. All patients were recruited and diagnosed by movement disorders specialists at Rush University Medical Center according to established criteria: diagnosis of PD based on the United Kingdom Parkinson’s Disease Society Brain Bank criteria [16], diagnosis of probable MSAp based on criteria from the American Academy of Neurology and American Autonomic Society [17], and diagnosis of probable PSP based on the NINDS-PSP criteria [14]. Healthy participants were recruited by advertisements in the Chicagoland area and were matched at group level for age, sex, and handedness. All participants were tested between 7∶30 AM and 12∶30 PM.

### Force Data Acquisition

**Figure 1:**
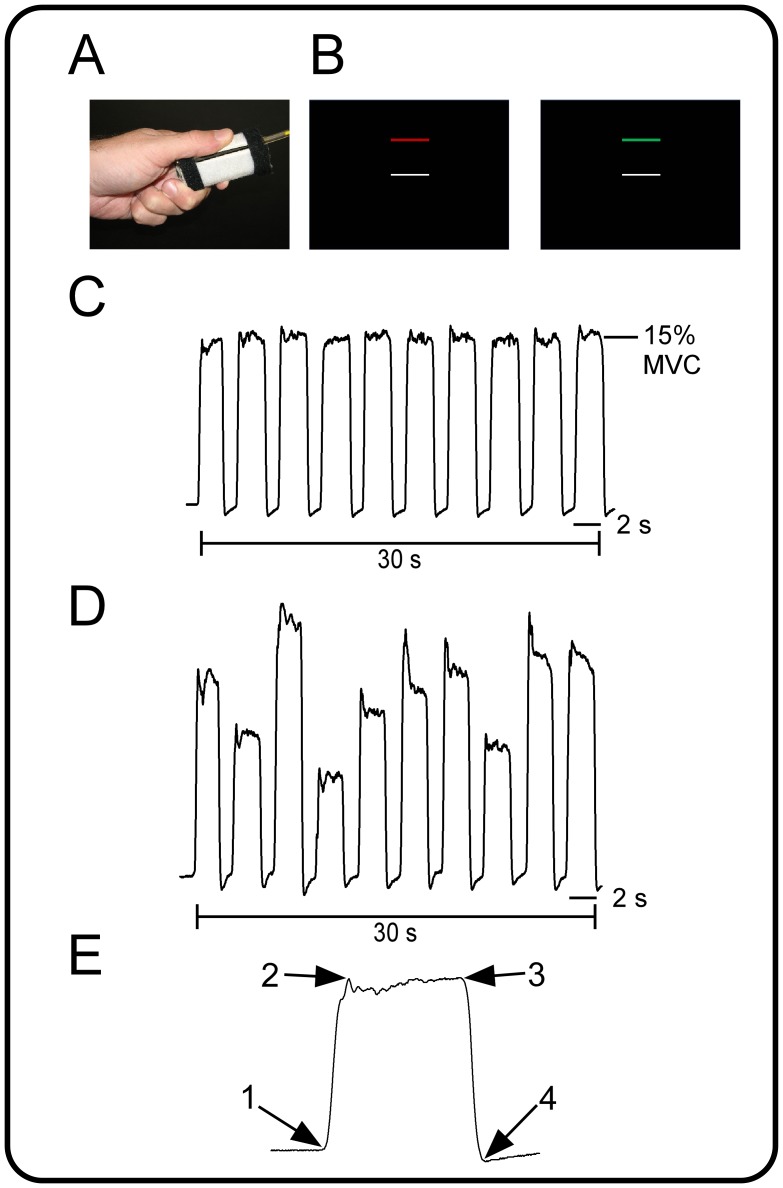
Participants completed two precision grip force tasks. A: the precision grip apparatus held with a modified grip. B: the visual display contained two horizontal bars presented against a high contrast black background. The target bar (red/green) was stationary during each force pulse, whereas the white force bar moved to provide online visual feedback. In both force tasks, participants produced 10, 2 s force pulses separated by 1 s of rest. C: in the SAME task, target amplitude was 15% of the participant’s MVC on all force pules. D: in the DIFF task, the target amplitude varied unpredictably from pulse to another. E: four time-points were determined for each force pulse as shown here. Arrow 1 marks the onset of force. Arrow 2 marks the onset of the steady force interval and arrow 3 marks the end of the steady force interval. Arrow 4 marks force offset. The rate of force increase is the slope of the black line between arrows 1 and 2. The duration of the force pulse is the time between arrows 2 and 3. The rate of force decrease is the slope of the black line between arrows 3 and 4. Mean force is calculated as the average force output between arrows 2 and 3. Variability of force output is the standard deviation of mean force.

Participants produced force against a custom-designed Bragg grating fiber-optic force transducer. The transducer was housed in a precision grip apparatus held between the thumb and index finger in a modified precision grip (Figure 1A). The force transducer and its housing were constructed from rigid, nonmetallic materials. The force transducer was calibrated and had a resolution of 0.025 N. Force data were digitized at 125 Hz using the si425 Fiber Optic Interrogator (Micron Optics, Atlanta, GA) and were collected and converted to Newtons (N) with customized software written in LabVIEW (National Instruments, Austin, TX). Force data were filtered online using a fourth-order dual-pass Butterworth filter with a low-pass frequency of 20 Hz.

### Experimental Procedures

All patients were rated by a movement disorders neurologist on the Unified PD rating scale (UPDRS) and were tested after overnight withdrawal from their anti-Parkinsonian medication. Patients were matched according to their off-medication UPDRS part III score. PD patients were not assigned a motor subtype (i.e., tremor-dominant, tremor-nondominant, and postural instability and gait difficulties) [18]; however, inspection of UPDRS-III scores suggests that 3 of 12 PD patients could be viewed as tremor-dominant. The classification of the remaining 9 PD patients was indeterminable because ratings for tremor items and posture/gait items were similar.

Each participant’s maximum voluntary contraction (MVC) was measured using a Jamar Hydraulic Pinch Gauge. All participants completed a training session to become familiar with the task. In the training and experimental sessions, patients produced force with their most affected hand.

As shown in Figure 1B, participants were provided with online visual feedback about their force [19]. The visual display contained two horizontal bars presented against a black background: a fixed target bar and a moveable white force bar. During all rest periods, the white force bar was stationary and the target bar was red. Participants were instructed to produce force when the target bar changed from red to green. Participants received online visual feedback via the moveable white force bar. No participants expressed difficulty differentiating between red and green.

Participants completed two tasks: (1) same amplitude (SAME) and (2) different amplitude (DIFF). In the SAME task, the target amplitude was always 15% of the participant’s MVC (Figure 1C). In the DIFF task, the target amplitude varied unpredictably from one pulse to the next (Figure 1D). The target amplitudes in the DIFF task were selected such that the average amplitude across pulses was 15% MVC. In both tasks, participants were instructed to produce force for 2 seconds. Each 2-second force pulse was separated by 1 second of rest, which was cued by a color change of the target bar from green to red. A series of 10 force pulses plus rest were completed to achieve a block of 30 seconds. Participants completed four blocks of grip force. Each task began and ended with 30 seconds of rest. The order of tasks was selected at random.

### Cognitive Tests

We conducted four cognitive tests: Mini-Mental State Exam (MMSE), Stroop task, Brief Test of Attention (BTA), and Digit Span. These measures were collected from all healthy individuals, PD patients, and MSAp patients, and 7 of 8 PSP patients during the same session as the motor control testing (off medications). We report the raw scores for the MMSE and the BTA and the summed score (forward+backward) for the Digit Span. We report the Stroop interference score, which captures performance in all three subsets of the test and adjusts for number of years of education.

### Force Data Analysis

Data analysis procedures were consistent with the methodology developed in previous work from our laboratory [20]. Visual inspection of force output was performed and four time-points were determined for each force pulse: onset of force, beginning and end of force production, and offset of force (Figure 1E). Six dependent measures were calculated: mean force, standard deviation of force, duration of the force interval, mean rate of change during the ramp up to the target, and mean rate of change during the decrease to baseline. These measures were calculated for each force pulse and then averaged across the force pulses to provide six mean dependent measures for each task and participant. All calculations were conducted with custom algorithms in MATLAB.

In addition, the number of force pulses produced was determined. In some cases, participants produced more than the instructed 10 pulses, both within and beyond the force interval. These pulses were included in the total number of pulses for the previously cued force interval. The number of pulses produced was tallied for each block and then averaged across the four blocks to provide a mean for each task and participant.

### Statistical Analysis

The first goal was to examine measures of grip force between groups. Separate one-way, univariate analyses of variance (ANOVA) for group (healthy control, PD, MSAp, PSP) were used for age, education, MVC, UPDRS Part III, Hoehn and Yahr stage, and disease duration. We conducted multivariate analysis of variance (MANOVA) for the force output measures using a 4 (group: healthy control, PD, MSAp, PSP) by 2 (task: SAME, DIFF) MANOVA. Cognitive measures were evaluated with a one-way MANOVA for group (healthy control, PD, MSAp, PSP). When a significant effect of group was observed, individual dependent variables were examined with univariate ANOVAs. Subsequently, Tukey’s HSD post hoc tests were conducted to determine if the mean difference between two groups was significant. All statistical tests were evaluated at an alpha of.05.

All dependent variables for which a significant effect of group was observed were entered into a ROC analysis. For measures that yielded an area under the curve (AUC)≥0.90, we report the cutoff value that differentiated the two groups at the highest levels of sensitivity and specificity. For dependent variables with AUC≥0.90, combination variables were calculated. Binary logistic regression in IBM SPSS Statistics (IBM, Chicago, IL) created predicted probabilities for combinations of two variables.

## Results

### Participants

The severity of Parkinsonism (Table 1) ranged from a UPDRS Part III motor score of 14 to 57. Across patient groups, the UPDRS score was not different, p>.05; however, disease duration was different, F(2, 29) = 13.20, p<.001. Tukey’s HSD post hoc tests demonstrated that patients with PD had longer disease duration than patients with MSAp and PSP. The results for age revealed an effect of group, F(3, 39) = 4.44, p = .009, and post-hoc tests showed that PSP patients were older than patients with PD, MSAp, and controls. Hoehn and Yahr stage scores were not equally distributed within groups, thus we used the Kruskal-Wallis H-test to test for differences across groups. The results revealed an effect of group, H(2) = 14.49, p = .001, and post-hoc tests showed that PD patients had a lower Hoehn and Yahr stage compared to MSAp and PSP.

**Table 1:** Subject characteristics and performance on cognitive tasks.

Variables	Group				Significant group differences
	PD	MSAp	PSP	Control	
Sample size	12	12	8	12	
Females	3	5	4	5	
Right-handed	11	10	8	11	
Right-hand tested	5	7	5	8	
Age, years	63.6 (7.4)	63.4 (8.7)	72.6 (5.6)	61.2 (8.8)	PSP>PD, MSAp, HC
Education, years	16.0 (2.3)	16.8 (3.9)	13.4 (3.6)	17.8 (2.9)	PSP<PD, MSAp, HC
MVC	65.8 (11.2)	57.9 (24.6)	42.9 (19.4)	70.3 (14.7)	PSP<PD, HC
UPDRS Part III	29 (22–37)	41 (14–57)	29 (17–39 )	n/a	*ns*
Hoehn & Yahr stage	2 (2–3)	3 (2–5)	3 (3–4)	n/a	PSP<PD, MSAp
Disease duration, months	75.7 (55.2)	7.2 (6.4)	14.3 (16.0)	n/a	PD>MSAp, PSP
Stroop Interference Score	−3.42 (5.53)	−1.25 (8.36)	0.14 (7.54)	0.75 (9.27)	*ns*
MMSE	29.33 (0.89)	27.25 (2.45)	25.00 (3.16)	28.75 (1.71)	PSP<PD, MSAp, HC
BTA	17.25 (3.33)	14.50 (3.78)	9.00 (4.69)	17.42 (2.31)	PSP<PD, MSAp, HC
Digit Span	18.17 (3.46)	17.42 (3.20)	13.43 (5.44)	18.25 (3.08)	PSP<PD, MSAp, HC

Values reported are sums, mean (SD), or median (range). Abbreviations: BTA = Brief Test of Attention; HC = healthy control; MMSE = Mini-Mental State Examination; MSAp = parkinsonian variant of multiple system atrophy; MVC = maximum voluntary contraction; *ns* = not significant; PD = Parkinson’s disease; PSP = progressive supranuclear palsy; UPDRS = Unified Parkinson’s Disease Rating Scale.

### Grip Force

**Figure 2:**
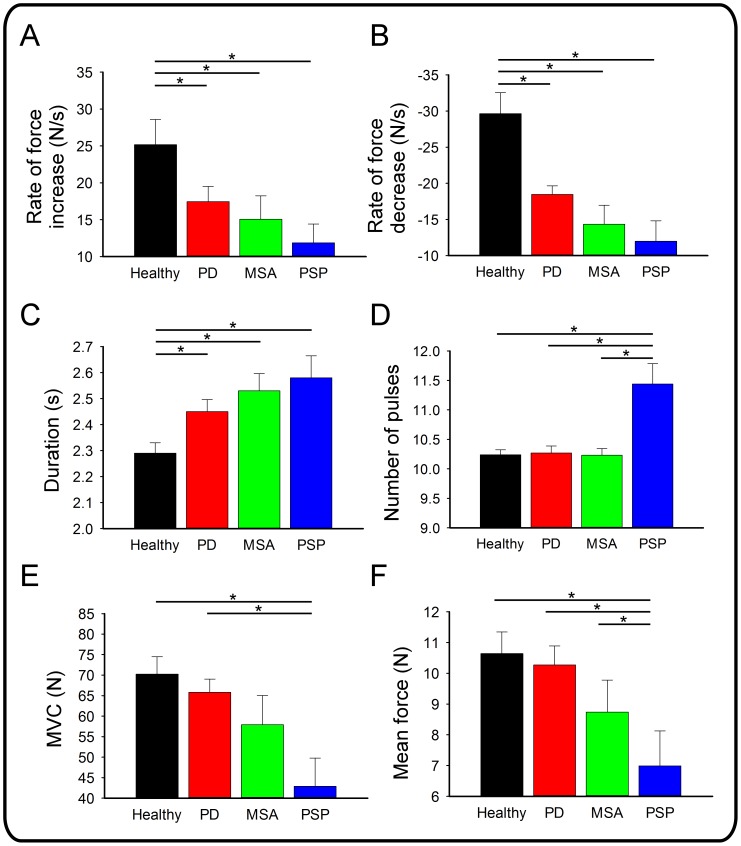
Means for force output variables for the healthy and patient groups. Error bars represent standard error of the mean. Asterisks (*) identify a significant mean difference at an alpha level of.05. A: Mean rate of force increase (N/s) for each group. B: Mean rate of force decrease (N/s) for each group. C: Mean duration of force pulse (s) for each group. D: Mean number of pulses for each group. E: Mean maximum voluntary contraction (MVC) in Newtons (N) for each group. F: Mean force output (N) for each group.

The MANOVA for grip force measures revealed an overall effect for group, Wilks’ λ = 0.206, F(24.00, 212.32) = 6.40, p<.001, partial eta squared = .409 (power = 1.00). There was no main effect or interaction involving task, ps >.300. Figure 2A shows that the rate of force increase was significant for group, F(3, 80) = 6.92, p<.01, such that PD, MSAp, and PSP were slower to increase force relative to controls. Figure 2B shows that the rate of force decrease was significant for group, F(3, 80) = 17.05, p<.001, and was due to PD, MSAp, and PSP decreasing force at slower rate compared to controls. The mean duration of force pulse revealed an effect of group F(3, 80) = 9.09, p<.001, such that PD, MSAp, and PSP produced longer force pulses relative to controls (Figure 2C). The group comparison for the number of pulses was also significant, F(3, 80) = 21.47, p<.001: patients with PSP produced more pulses than any other group (Figure 2D).

The analysis of MVC revealed a difference across groups, F(3, 40) = 4.14, p = .012, such that patients with PSP were weaker than patients with PD and controls (Figure 2E). It follows that mean force output yielded a main effect of group, F(3, 80) = 6.17, p = .001 (Figure 2F), such that PSP produced lower mean force relative to PD and MSAp as well as controls. The analysis of force in percent MVC provides an index of force output relative to the target and was not different across groups, p>.100. The standard deviation of force in percent MVC was different across groups, F(3, 80) = 6.08, p = .001, such that MSAp and PSP were more variable than PD. MSAp and PSP were more variable than controls, however, these comparisons did not reach significance (ps >.054).

### Cognitive Measures

The MANOVA for cognitive measures revealed a significant effect for group, Wilks’ λ = 0.389, F(12.00, 92.89) = 3.32, p<.001, partial eta squared = .270 (power = 0.980). The effect of group was examined by individual ANOVAs, which revealed an effect of group for the BTA, F(3, 38) = 14.74, p<.001; MMSE, F(3, 38) = 5.25, p = .004; and Digit Span, F(3, 38) = 4.75, p = .007. Posthoc tests demonstrated an identical pattern of results: PSP scored lower than PD, MSAp, and controls. In contrast, the results for the Stroop Interference Score, F(3, 38) = 0.68, p = .416, did not approach significance.

### ROC Analysis

Table 2 reports the contrasts for which AUC≥0.90. Patients with PSP were discriminated from healthy individuals by the rate of force decrease, the number of pulses produced, and BTA score. Patients with PSP were discriminated from patients with PD by the number of pulses produced and scores on the MMSE and BTA. Lastly, patients with PSP were discriminated from patients with MSAp by a single variable, the number of pulses produced. Notably, for the contrasts between MSAp and PD and MSAp and healthy individuals, none of the cognitive or motor variables yielded an AUC≥0.90.

**Table 2:** Receiver operating characteristic (ROC) curves for (A) individual and (B) combined variables.

Classification state	Variable	AUC	Cutoff	Specificity	Sensitivity
A. ROC results for individual variables					
PSP v. Healthy	Rate of force decrease	0.906	−17.90	91.7%	87.5%
PSP v. Healthy	Number of pulses	0.990	10.56	100%	87.5%
PSP v. Healthy	BTA	0.938	13.5	91.7%	87.5%
PSP v. PD	Number of pulses	0.948	10.81	91.7%	75.0%
PSP v. PD	MMSE	0.952	27.5	100%	87.5%
PSP v. PD	BTA	0.935	11.5	91.7%	87.5%
PSP v. MSAp	Number of pulses	0.964	10.56	91.7%	87.5%
B. ROC results for combined variables					
PSP v. Healthy	BTA+number of pulses	1.000	n/a	100%	100%
PSP v. PD	BTA+number of pulses	1.000	n/a	100%	100%
PSP v. MSAp	BTA+number of pulses	0.994	n/a	85.7%	100%

Abbreviations: AUC = Area under the curve; BTA = Brief Test of Attention; MSAp = parkinsonian variant of multiple system atrophy; MMSE = Mini-mental state exam; PD = Parkinson's disease; PSP = progressive supranuclear palsy.

Since number of pulses and BTA were the variables that were consistently useful in distinguishing PSP from health and from PD, we evaluated whether the combination of these two variables would better discriminate PSP from PD, MSAp, and controls. Indeed, as shown in Table 2B, BTA+number of pulses discriminated PSP from PD, MSAp, and controls with high degrees of sensitivity and specificity. Since this combination variable was successful in distinguishing PSP from the other patients, we evaluated whether the combination variable would distinguish MSAp from PD or controls. The results demonstrated that this combination variable was not useful in distinguishing MSAp from PD or health (highest AUC = 0.747).

## Discussion

The present study examined grip force control in healthy individuals and patients with PD, MSAp, and PSP. All patients were slower at contracting and relaxing force relative to healthy individuals. Patients with PSP produced more force pulses than all other groups, suggesting that this deficit may be specific to PSP. In addition to examining force control, we examined several cognitive measures. PSP patients showed greater cognitive impairment relative to all other groups. A key finding was that combining force control and cognitive variables led to improved patient discrimination.

Consistent with previous work, PD was characterized by a slower rate of force development [6], [7], [8], [9], [10] and force relaxation [8], [11], [12]. We extend these findings to show that deficits in rapidly increasing and decreasing force are also characteristic of MSAp and PSP. Further, the average duration of force pulses was longer for all patient groups relative to controls. Taken together, these measures of grip force may comprise a common behavioral feature of Parkinsonism, wherein patients have difficulty modulating the rate of change and the duration of force output. This commonality may reflect the fact that PD, MSAp, and PSP have pathology in the basal ganglia [21], [22], [23]. Indeed, fMRI studies have shown that basal ganglia activity is related to bradykinesia [24] and subthalamic nucleus deep brain stimulation improves the rate of grip force development [25], [26] and bradykinesia [27] in patients with PD.

A novel finding was that patients with PSP produced more force pulses than other groups. This phenomenon may reflect a cognitive impairment and/or impaired motor inhibition. PSP patients were impaired on measures of auditory divided attention (BTA), verbal working memory (Digit Span), and general cognition (MMSE). Patients with PSP develop frontal cognitive impairment and undergo faster cognitive decline relative to PD and MSAp [15]. Frontal cognitive impairment can include slowness of thinking and responding, as well as impaired attention, set shifting, and categorization [14], [15]. Increased slowness of thinking and responding may explain why the PSP patients in our study continued to perform the motor task after being visually cued to stop [14], [15]. An alternate hypothesis is that PSP patients have difficulty inhibiting the production of force once it has begun. Indeed, cognitive decline in PSP is linked to neurodegeneration in the frontal cortex, an area well-known for its role in action inhibition [28], [29]. The patients in our study had similar UPDR-III scores. Given that the UPDRS-III does not include examination of motor inhibition, it may be useful to perform a clinical test of motor inhibition to differentiate PSP from other Parkinsonian disorders. One such test is the “applause sign” - the inability to stop clapping after being asked to imitate the examiner’s three handclaps [30], [31]. However, the applause sign is not specific for PSP and may generalize to other movement disorders [32], [33], [34]. One limitation of the applause sign is that it does not account for muscle activity that does not result in a clap. The task in the current study measures small changes in force output and therefore may be more sensitive to differences between patient groups.

Repetitive tapping and handwriting tasks also provide helpful clues to distinguish PSP from PD [35]. In particular, PSP patients have smaller finger separation amplitudes relative to PD patients during a repetitive tapping task. Further, finger separation amplitudes remain constant in PSP, whereas amplitudes are consistently reduced in PD. Similarly, micrographia and lack of decrement in handwriting size are more common in PSP than in PD [35]. These findings, in combination with the current work, demonstrate that bedside behavioral assessments may identify distinctive features of PSP. More important, the current work demonstrates that the combination of behavioral and cognitive assessments is more useful in distinguishing PSP from PD and MSAp than the use of each assessment independently.

One of the greatest challenges when comparing forms of Parkinsonism is to obtain samples that are well-matched for both disease severity and age. Disease progression is more rapid and age of onset is older for MSAp and PSP compared to PD. Given that the purpose of this study was to examine motor and cognitive deficits across groups, it was important to match the groups for disease severity, even though this meant the PSP patients were older than patients in the other groups. Since age is known to affect strength [36], [37], [38], this may have contributed to the observed weakness in MVC found in the PSP group. Further, since low forces are the most variable [39], [40], [41], [42], the weakness in MVC may have contributed to the increased variability found in the PSP group.

The current work represents a first step in identifying tools that help distinguish MSAp and PSP from PD. This type of work relies on patients who have been given a probable diagnosis, which in turn limits the findings to patients who have a diagnosis. A logical next step is to employ the most useful tools from this study (i.e., BTA, MMSE, and number of pulses) in a longitudinal study following early-stage patients with a less-certain diagnosis. Such work could identify the ability of each tool (or combination of tools) to distinguish patients at different stages of the disease. Future studies should aim to increase the sample size and to broaden the distribution of age, disease severity, and disease duration. Another interesting question that arises from this work is whether the same motor and cognitive tests can distinguish MSAp and PSP from different motor subtypes of PD (i.e., tremor-dominant and postural instability/gait difficulties) [18].

There are two notable caveats to this study. First, we recognize that our patient groups are small. Future studies should use these techniques in a larger sample. Second, medication could have lingering effects after overnight withdrawal. For ethical and safety reasons, this study was limited to overnight withdrawal from anti-Parkinson medication. Moreover, because PD patients are typically more responsive to medication than MSAp and PSP patients, PD patients are often taking more medication than MSAp and PSP patients. The cleanest solution to this problem is to study drug-naïve patients; however, such patients often do not have a probable diagnosis. Another approach is to study patients on medication. Since PD responds to anti-Parkinson medications and MSAp and PSP typically do not respond as well, greater disparity between patient groups may be evident when PD patients are taking their medication.

One of the most notable findings was that a combination of the number of pulses and BTA score distinguished PSP from with PD, MSAp, and controls. Novel tools that distinguish these patients are important because they provide testable hypotheses related to the pathophysiology of PSP. Other tools that have shown diagnostic promise include olfactory testing [43], [44], neuropsychological tests, α-synuclein concentration in cerebrospinal fluid [45], [46], magnetic resonance and diffusion weighted imaging [47], [48], [49], [50], [51], and single photon emission tomography (SPECT) of the dopamine system [52], [53]. Future studies may be available that compare these approaches in distinguishing PD from atypical Parkinsonism.
